# Cooccurrence of Antibiotic Resistance and Hypervirulence in High-Risk Carbapenem-Resistant K14.K64 and Wzi209 Klebsiella pneumoniae Strains Driven by Plasmids and Their Derivatives

**DOI:** 10.1128/spectrum.02541-21

**Published:** 2022-08-22

**Authors:** Weinan Zhu, Ying Liu, Feng Chen, Shiyu Chen, Yongqiang Zhu, Hu Li, Jiawei Wang, Jingxian Liu, Yuanrui Li, Jiajia Yu, Hongyan Guan, Jing Yu, Lisong Shen

**Affiliations:** a Department of Laboratory Medicine, Xin Hua Hospital, Shanghai Jiao Tong University School of Medicine, Shanghai, China; b Shanghai-MOST Key Laboratory of Health and Disease Genomics, Chinese National Human Genome Center at Shanghai, Shanghai, China; c Medical Laboratory Sciences, Shanghai Jiao Tong University School of Medicine, Shanghai, China; d Xin Hua Children’s Hospital, Shanghai Jiao Tong University School of Medicine, Shanghai, China; University of Pittsburgh School of Medicine

**Keywords:** carbapenem-resistant *Klebsiella pneumoniae*, plasmids, antibiotic resistance, hypervirulence, K14.K64, wzi209, coevolution

## Abstract

Emerging hypervirulent carbapenem-resistant Klebsiella pneumoniae (hv-CRKP) is a severe public health problem worldwide. To assess the cooccurrence of CRKP and hv-CRKP, a total of 1,181 CRKP isolates were collected from 2009 to 2018, covering their initial occurrence to outbreaks. Overall, two major capsular serotypes, namely, wzi209-CRKP and K14.K64-CRKP, were identified as being prevalent in pediatric and adult patients, respectively. Most isolates carried *bla*_KPC_, and the *bla*_KPC_-carrying hybrid plasmid IncFII-IncR, which was stable and transferable, was identified. The conjugation region (*traN*/*traC*) of IncFII-IncR was found to be variable, and the genes were used as markers to identify the transmission of strains among patient groups in this study. Notably, hv-CRKP was characterized by screening for four virulence genes (*rmpA*, *iroN*, *terW*, and *rmpA2*) in all 977 *bla*_KPC_-carrying K14.K64-CRKP and wzi209-CRKP strains. Two virulence types, namely, *rmpA*/*iroN*/*terW*/*rmpA2* positive and *terW*/*rmpA2* positive, were found. The corresponding virulence plasmids Vir1, i.e., nonconjugative IncFIB(k)-IncHI1B, and Vir2, i.e., conjugative antibiotic-resistant IncFIB-IncHI1B, were further characterized. Both Vir1 and Vir2 were stable, and the transferability of Vir2 was significantly higher than that of IncFII-IncR. However, none of the Vir1- or Vir2-carrying strains exhibited the hypervirulent phenotype. Meanwhile, hv-CRKP (*terW*/*rmpA2* positive) was found in late 2018 among wzi209-CRKP strains. The corresponding Vir2-related fragment was characterized as chromosomally integrated, which dramatically enhanced the virulence of wzi209-CRKP. Transmission of hv-CRKP among patient groups was also confirmed according to virulence elements. Taken together, CRKP and hv-CRKP occurred on a large scale. Plasmids and their derivatives played an important role on this process. Surveillance and intervention of hv-CRKP are urgently needed.

**IMPORTANCE** Currently, an increasing number of hv-CRKP strains have been reported and pose a substantial threat to public health worldwide, because these strains are considered to be simultaneously hypervirulent, carbapenem resistant, and transmissible. In this study, we provided a complete transition process of CRKP and hv-CRKP from their early emergence to outbreak in 10 years. We identified two epidemic groups, K14.K64 (wzi64)-CRKP and wzi209-CRKP, in adult and pediatric patients, respectively. K14.K64 (wzi64)-CRKP was widely present, while wzi209-CRKP was rarely reported as an epidemic type. We discovered a large scale of hv-CRKP transmission from CRKP and determined the importance of antibiotic resistance and virulence plasmids and their derivatives for the transition of CRKP and hv-CRKP. Two virulence plasmids coexist in out hospital, but neither of them enhanced virulence. Notably, we found a newly emerged type of CRKP, hypervirulent wzi209-CRKP, which had dramatically enhanced virulence, making it a great threat to human health.

## INTRODUCTION

Carbapenem-resistant Klebsiella pneumoniae (CRKP) has become the major class of bacterial pathogens that pose a significant threat to global public health (1–4). KPC-2-producing Klebsiella pneumoniae has emerged as a leading cause of hospital outbreaks worldwide, and a significant proportion of the KPC burden in China is associated with a single Klebsiella pneumoniae multilocus sequence typing sequence type (ST), ST11 (5–7). KPC-2-carrying antibiotic resistance plasmids were reported to contribute to the rapid dissemination of CRKP. Since the first case was reported in Zhejiang, cases of CRKP have been identified in almost every region of China (5, 8, 9).

Of great concern, hypervirulent CRKP (hv-CRKP) infections have been increasingly reported, which indicates a worrying convergence of carbapenem resistance and hypervirulence in an already epidemic lineage of Klebsiella pneumoniae (10–13). Several studies suggested that hv-CRKP had enhanced virulence and acquisition of carbapenem resistance, although the incidence reported was still low (11, 14–17). Virulence plasmids harboring capsular polysaccharide regulator genes (*rmpA* and *rmpA2*) and several siderophore gene clusters were identified to be related to the hypervirulence phenotype (18–21). The transmission of antibiotic-resistant and virulent plasmids among CRKP and hv-CRKP strains generated the potential “superbug” (22–25).

To date, research on the dissemination and transmission of epidemiologically successful CRKP has been limited. In this study, we detected the antibiotic resistance and hypervirulence of CRKP and hv-CRKP in a tertiary hospital in China in 2009 to 2018. The CRKP and hv-CRKP occurred on a large scale, mediated by plasmids and their derivatives, and hypervirulent wzi209-CRKP was identified to have extremely high virulence and can be considered to be a newly emerged threat to public health. This investigation was performed on antibiotic resistance and virulence simultaneously to better provide insight into nosocomial infection control and clinical antimicrobial therapy for CRKP and hv-CRKP.

## RESULTS

### wzi209-CRKP and K14.K64-CRKP prevalence among pediatric and adult patients.

A total of 1,181 CRKP strains were collected from individual inpatients from 2009 to 2018. The amount of CRKP has dramatically increased over time since 2014. A total of 53 capsular types were found by *wzi* typing. Notably, two major *wzi* types, K14.K64 (wzi64) and wzi209, appeared, accounting for 48.69% and 36.16%, respectively (Table 1; also see Fig. S1A in the supplemental material). The first wzi209-CRKP strain was isolated in 2010, earlier than K14.K64-CRKP, which was isolated in 2013. Furthermore, 96.35% of K14.K64-CRKP strains were isolated from adult patients, and 3.65% were isolated from pediatric patients. Meanwhile, 26.23% of wzi209-CRKP strains were isolated from adult patients, and 73.77% were isolated from pediatric patients (see Fig. S1B and Table S1). Notably, K14.K64-CRKP strains isolated from adult patients and wzi209-CRKP strains isolated from pediatric patients were found to have mainly contributed to the hospital-wide dissemination over time. Clonal shift indeed occurred in adult patients, from wzi209-CRKP to K14.K64-CRKP, while wzi209-CRKP continued to be the major type disseminated in pediatric patients (see Fig. S1C). Moreover, the majority of non-K14.K64/wzi209-CRKP strains were isolated from pediatric patients, who contained more capsular types (see Fig. S2A and B). Both capsular types and amounts of non-K14.K64/wzi209-CRKP strains showed huge increases in 2015, which mainly occurred among pediatric patients (see Fig. S2C).

**TABLE 1:** Prevalence of CRKP and *bla*_KPC_-carrying CRKP strains isolated in 2009 to 2018

CRKP type	No. of isolates										
	2009	2010	2011	2012	2013	2014	2015	2016	2017	2018	Total
CRKP	1	6	28	9	35	64	142	271	264	361	1181
K14.K64					14	50	68	142	139	162	575
K14.K64-CRKP-adult					14	50	65	139	127	159	554
K14.K64-CRKP-pediatric							3	3	12	3	21
wzi209		3	26	8	19	6	37	89	91	148	427
wzi209-CRKP-adult		3	26	7	12	4	21	13	14	12	112
wzi209-CRKP-pediatric				1	7	2	16	76	77	136	315
Non-K14.K64/wzi209-CRKP	1	3	2	1	2	8	37	40	34	51	179
Non-K14.K64/wzi209-CRKP-adult	1	2		1	2		2	7	5	22	42
Non-K14.K64/wzi209-CRKP-pediatric		1	2			8	35	33	29	29	137
*bla*_KPC_-CRKP		4	26	8	35	57	104	245	223	321	1023
K14.K64					14	50	64	139	125	159	551
K14.K64-CRKP-adult					14	50	63	136	113	157	533
K14.K64-CRKP-pediatric							1	3	12	2	18
wzi209		3	26	8	19	6	37	89	91	147	426
wzi209-CRKP-adult		3	26	7	12	4	21	13	14	12	112
wzi209-CRKP-pediatric				1	7	2	16	76	77	135	314
Non-K14.K64/wzi209		1			2	1	3	17	7	15	46
Non-K14.K64/wzi209-CRKP-adult		1			2		2	6	4	14	29
Non-K14.K64/wzi209-CRKP-pediatric						1	1	11	3	1	17

### *bla*_KPC_-carrying IncFII-IncR hybrid plasmid dissemination in K14.K64-CRKP and wzi209-CRKP.

**FIG 1:**
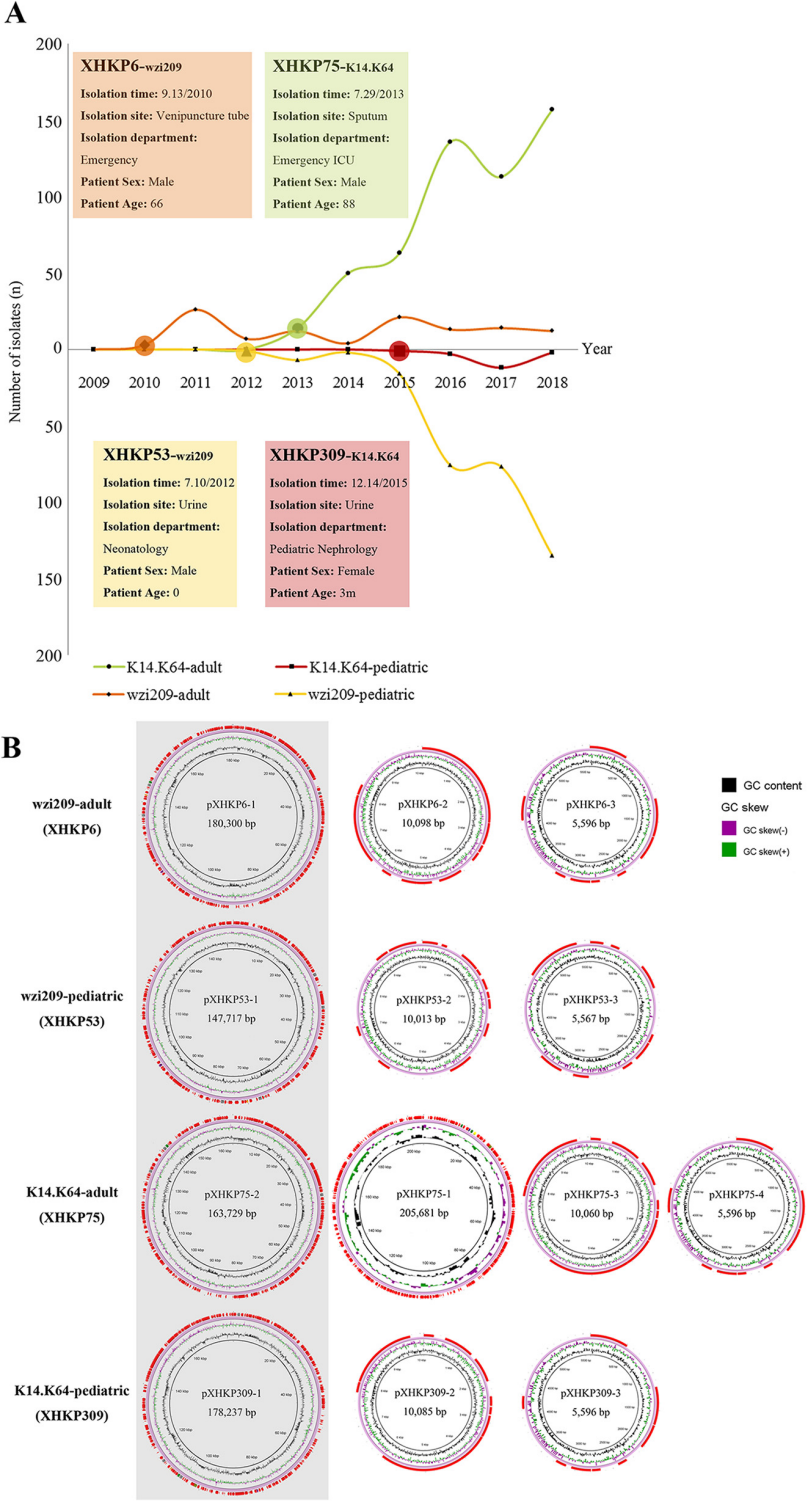
*bla*_KPC_-carrying K14.K64-CRKP and wzi209-CRKP strains disseminated in adult and pediatric patients and features of antibiotic resistance plasmids. (A) Timeline of *bla*_KPC_-carrying K14.K64-CRKP and wzi209-CRKP among adult and pediatric patients. The *x* coordinate is the year, and the *y* coordinate is the number of *bla*_KPC-2_-carrying isolates in each year. wzi209-CRKP-adult strains are colored orange, wzi209-CRKP-pediatric strains are colored yellow, K14.K64-CRKP-adult strains are colored green, and K14.K64-CRKP-pediatric strains are colored red. General information on XHKP6, XHKP53, XHKP75, and XHKP309 is listed. (B) Plasmid profiles of XHKP6, XHKP53, XHKP75, and XHKP309. *bla*_KPC-2_-carrying antibiotic resistance plasmids are shaded gray, and GC skew and GC content are marked.

A total of 86.6% of CRKP strains carried *bla*_KPC_, with 95.83% in K14.K64-CRKP, 99.77% in wzi209-CRKP, and 25.7% in non-K14.K64/wzi209-CRKP (Table 1). The first emerging strains of K14.K64-CRKP and wzi209-CRKP isolated from adult and pediatric patients, XHKP6, XHKP53, XHKP75, and XHKP309, were selected and sequenced with a Pacific Biosciences (PacBio) RS II system (Fig. 1A; also see Table S2). According to the whole-genome sequences, three plasmids were identified, including *bla*_KPC_-carrying IncFII-IncR with a size of ~170 kb and two small plasmids (a 10-kb ColRNAI and a 5-kb unknown plasmid). XHKP75 contained an extra antibiotic-resistant IncFIIk-IncFIBk hybrid plasmid with a size about 200 kb (Fig. 1B). As shown in Fig. S3 in the supplemental material, *bla*_KPC_-carrying IncFII-IncR in XHKP6, XHKP53, XHKP75, and XHKP309 (pXHKP6-1, pXHKP53-1, pXHKP75-2, and pXHKP309-1, respectively) showed very high similarity with pKPGD-4 (GenBank accession number NZ_CP025952.1) and pKPC2_020002 (GenBank accession number CP028541.2) and high identity with both pHN7A8 (GenBank accession number JN232517.1) and pKP048 (GenBank accession number FJ628167.2), which contained IncFII-pHN7A8-*rep* and IncR-*rep*, respectively.

### Conjugation fragments revealing the transmission of *bla*_KPC_-carrying IncFII-IncR among patient groups.

**FIG 2:**
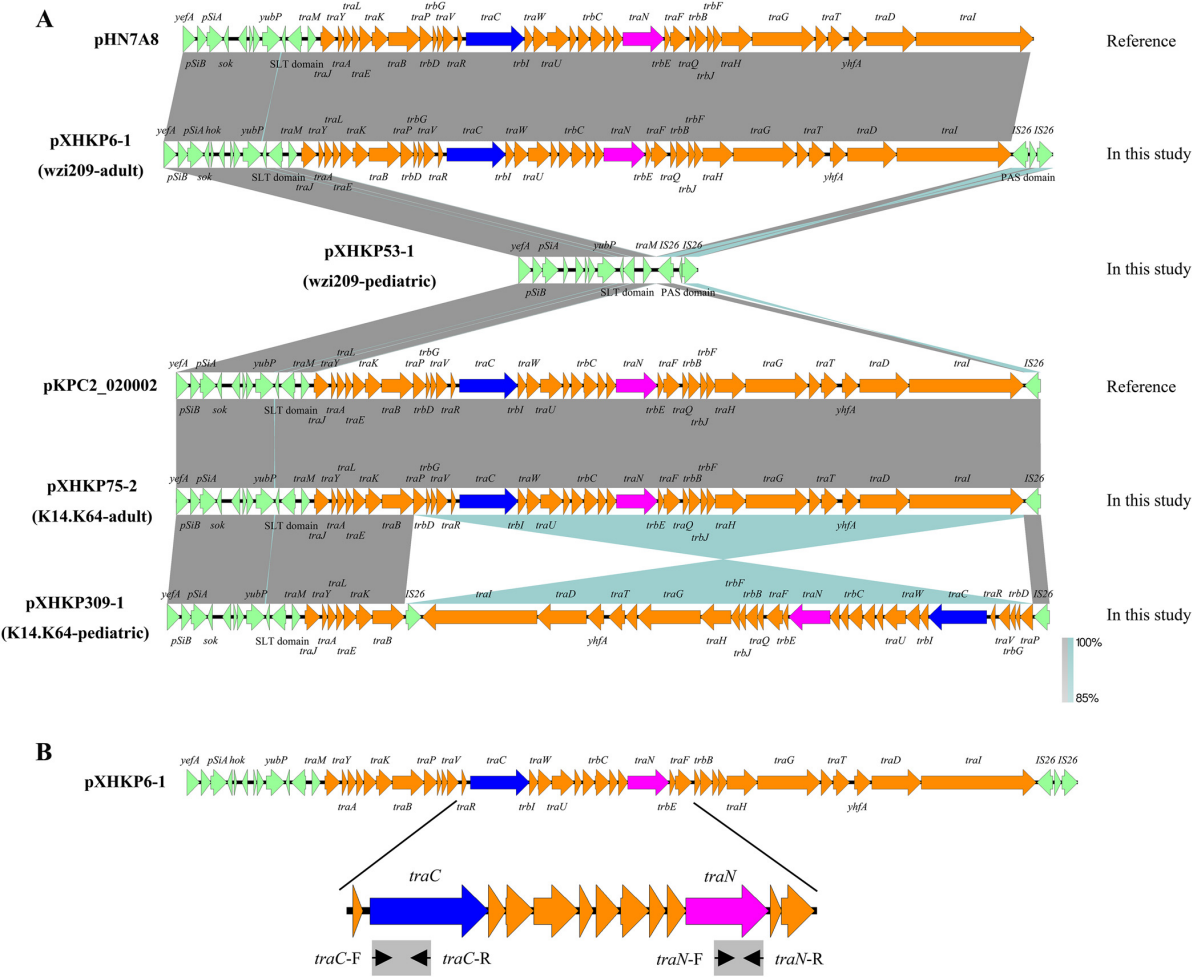
Sequence alignment and characterization of conjugation fragments in *bla*_KPC_-carrying plasmids. (A) Alignment of conjugation fragments in pXHKP6-1, pXHKP53-1, pXHKP75-2, pXHKP309-1, pHN7A8, and pKPC2_020002. Regions exhibiting sequence homology are shown in gray. Regions exhibiting the reverse direction are shown in dark green. The conjugation fragments are orange, regions on both sides of the conjugation fragments are green, *traC* is blue, and *traN* is purple. (B) Locations of primers used to target the conjugation fragments. Primers are marked with black triangles. The amplified fragments are shown in gray rectangles. The primer pair *traC*-F/*traC*-R was used to identify *traC*, and the primer pair *traN*-F/*traN*-R was used to identify *traN*.

Notably, a conjugation-related fragment with a size of ~32 kb was absent in pXHKP53-1 which made it smaller (Fig. 2A). To identify the conjugation fragment in *bla*_KPC_-carrying K14.K64-CRKP and wzi209-CRKP, PCR screening was performed with primers targeting *traC* and *traN* (Fig. 2B and Table 2; also see Table S5). As we know, this conjugative fragment was rarely found in wzi209-CRKP strains in pediatric patients here, which was consistent with the genomic features of XHKP53. Sporadic gain and loss happened over time.

**TABLE 2:** Prevalence of conjugation genes in *bla*_KPC_-carrying K14.K64-CRKP and wzi209-CRKP strains among adult and pediatric patients in 2009 to 2018

Conjugation genes and CRKP type	Proportion (%) (no. of isolates with genes/total no. of isolates)									
	2009	2010	2011	2012	2013	2014	2015	2016	2017	2018
*traN*										
K14.K64-CRKP-adult (483/533 isolates)					100.0 (14/14)	96.0 (48/50)	96.8 (61/63)	95.6 (130/136)	96.5 (109/113)	75.2 (118/157)
K14.K64-CRKP-pediatric (16/18 isolates)							100.0 (1/1)	66.7 (2/3)	100.0 (12/12)	50.0 (1/2)
wzi209-CRKP-adult (72/112 isolates)		100.0 (3/3)	80.8 (21/26)	85.7 (6/7)	66.7 (8/12)	75.0 (3/4)	76.2 (16/21)	46.2 (6/13)	28.6 (4/14)	41.7 (5/12)
wzi209-CRKP-pediatric (20/314 isolates)				0.0 (0/1)	0.0 (0/7)	0.0 (0/2)	18.8 (3/16)	15.8 (12/76)	3.9 (3/77)	1.5 (2/135)
*traC*										
K14.K64-CRKP-adult (476/533 isolates)					100.0 (14/14)	96.0 (48/50)	93.7 (59/63)	93.4 (127/136)	96.5 (109/113)	75.2 (118/157)
K14.K64-CRKP-pediatric (16/18 isolates)							100.0 (1/1)	66.7 (2/3)	100.0 (12/12)	50.0 (1/2)
wzi209-CRKP-adult (76/112 isolates)		100.0 (3/3)	80.8 (21/26)	85.7 (6/7)	75.0 (9/12)	50.0 (2/4)	76.2 (16/21)	76.9 (10/13)	28.6 (4/14)	41.7 (5/12)
wzi209-CRKP-pediatric (15/314 isolates)				0.0 (0/1)	0.0 (0/7)	0.0 (0/2)	0.0 (0/16)	15.8 (12/76)	1.3 (1/77)	1.5 (2/135)

**FIG 3:**
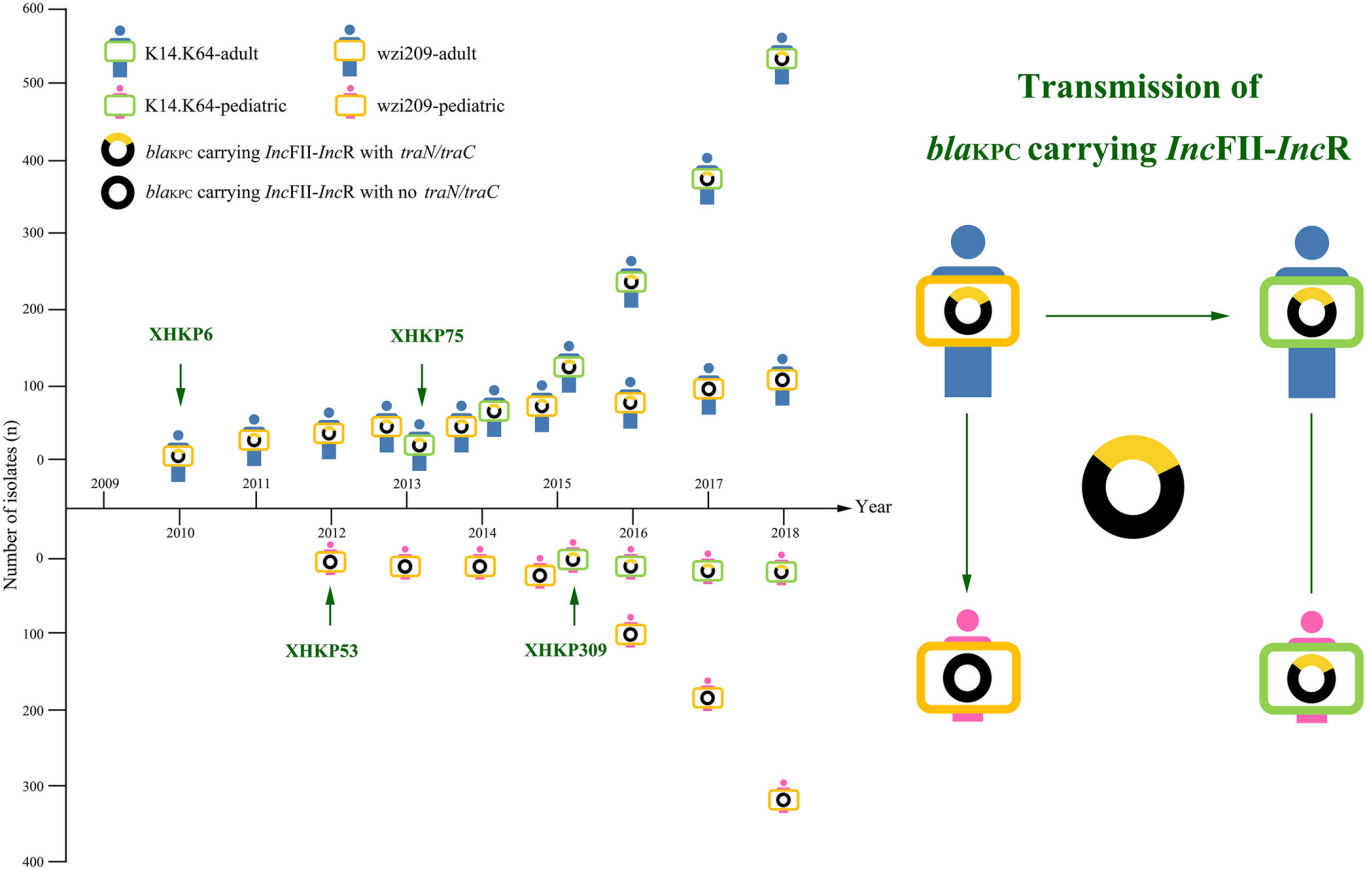
Dissemination of *bla*_KPC_-carrying wzi209-CRKP and K14.K64-CRKP among patient groups by IncFII-IncR and their derivatives. The *x* coordinate is the year, and the *y* coordinate is the accumulated number of isolates of each group. Adult patients are colored blue, and pediatric patients are colored pink. K14.K64-CRKP is marked in green, and wzi209-CRKP is marked in orange. K14.K64-CRKP-adult, K14.K64-CRKP-pediatric, wzi209-CRKP-adult, and wzi209-CRKP-pediatric are shown. *traN/traC* is marked in yellow on the *bla*_KPC_-carrying IncFII-IncR. Strains carrying various plasmids are shown. The isolation times for XHKP6, XHKP53, XHKP75, and XHKP309 are marked with green arrows. The transmission of *bla*_KPC_-carrying IncFII-IncR is shown. Green arrows represent determined transmission directions, while the green line without an arrow represents uncertain transmission direction.

As shown in Fig. 3 and Table 2, the first *bla*_KPC-2_-carrying IncFII-IncR, pXHKP6-1, was discovered in wzi209-CRKP-adult and was *traC*/*traN* positive. However, the first *bla*_KPC-2_-carrying IncFII-IncR in wzi209-CRKP-pediatric, pXHKP53-1, was *traC*/*traN* negative, which indicated that the missing event might have occurred on plasmids in the process of transmission of CRKP from wzi209-CRKP-adult to wzi209-CRKP-pediatric. Meanwhile, the first *bla*_KPC-2_-carrying IncFII-IncR plasmids in K14.K64-CRKP-adult, pXHKP75-2, and K14.K64-CRKP-pediatric, pXHKP309-1, were *traC*/*traN* positive. All of the aforementioned results indicated that IncFII-IncR in K14.K64-CRKP-adult was transmitted also from wzi209-CRKP-adult but not wzi209-CRKP-pediatric, while plasmid transmission to K14.K64-CRKP-pediatric was not confirmed.

### Outbreak of hv-CRKP among *bla*_KPC_-carrying K14.K64-CRKP and wzi209-CRKP.

**FIG 4:**
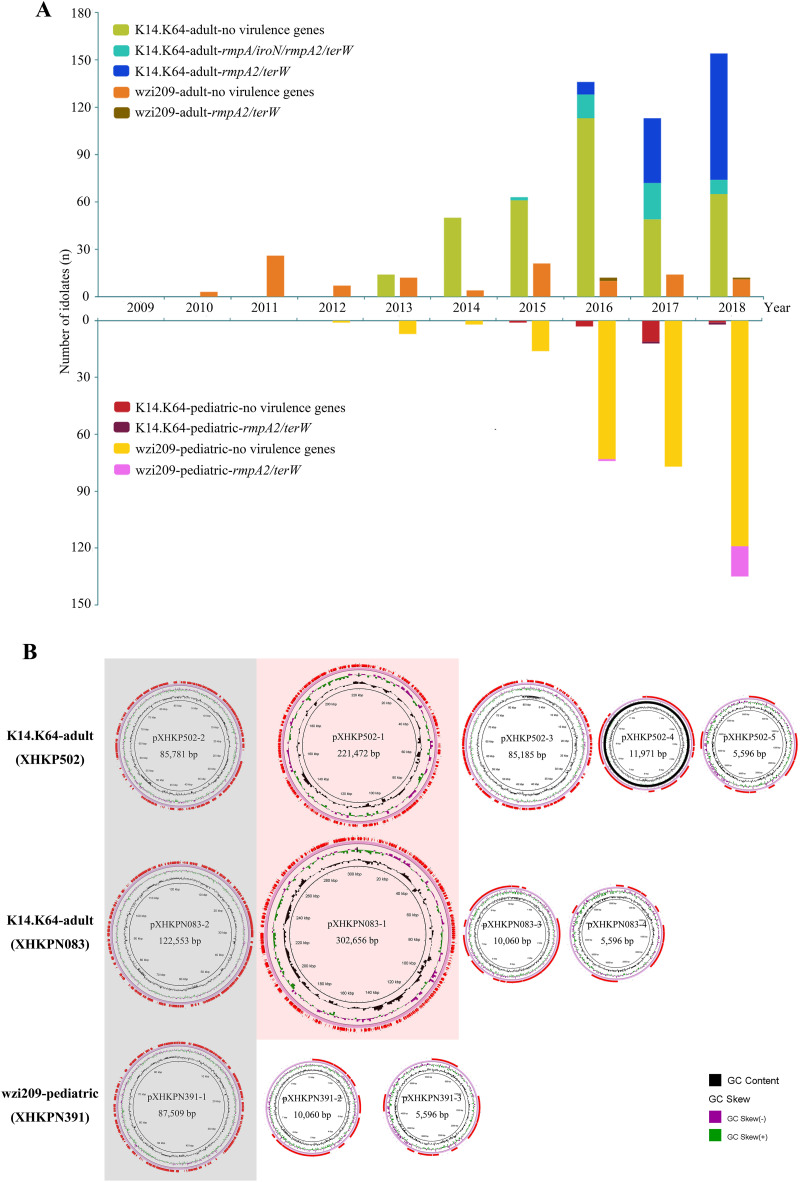
Prevalence of *bla*_KPC_-carrying K14.K64-CRKP and wzi209-CRKP with and without virulence genes from 2009 to 2018 and plasmid features of hv-CRKP. (A) Prevalence of virulence gene-carrying isolates in adult and pediatric patients. Different CRKP groups, including CRKP with no virulence genes, CRKP-*rmpA/iroN/rmpA2/terW*, and CRKP-*rmpA2/terW*, are shown. Primers are listed as reported previously. (B) Plasmid profiles of XHKP502, XHKPN083, and XHKPN391. *bla*_KPC-2_-carrying IncFII-IncR plasmids are shaded in gray. Virulence plasmids are in shades of red. GC skew and GC content are marked.

We detected four virulence genes, *rmpA*, *iroN*, *rmpA2*, and *terW*, in all 977 *bla*_KPC_-carrying K14.K64-CRKP and wzi209-CRKP isolates (see Tables S3 and S5). As shown in Fig. 4A and Table 3, hv-CRKP mainly appeared among K14.K64-CRKP-adult strains. In total, two different virulence types were clearly found; one was *rmpA*/*iroN*/*rmpA2*/*terW* positive and another was *rmpA2*/*terW* positive. Although the *rmpA*/*iroN*/*rmpA2*/*terW*-type strains appeared earlier, *rmpA2*/*terW*-type strains were more prevalent and became the major epidemic hv-CRKP type quickly. A few strains belonged to neither type and are not shown here. Actually, *rmpA2*/*terW*-type strains appeared first among both K14.K64-CRKP-adult and wzi209-CRKP-adult strains in 2016, but outbreaks occurred among K14.K64-CRKP-adult strains only later. Of note, *rmpA2*/*terW*-type strains also appeared among wzi209-CRKP-pediatric isolates, which were concentrated in late 2018 and which will likely to be the next epidemic type.

**TABLE 3:** Prevalence of two virulence types in *bla*_KPC-2_-carrying K14.K64-CRKP and wzi209-CRKP strains among adult and pediatric patients in 2009 to 2018

Virulence type and CRKP type	Proportion (%) (no. of isolates with virulence type/total no. of isolates)									
	2009	2010	2011	2012	2013	2014	2015	2016	2017	2018
*rmpA/iroN/rmpA2/terW*										
K14.K64-CRKP-adult (9.2% [49/533 isolates])					0 (0/14)	0 (0/50)	3.2 (2/63)	11.0 (15/136)	20.4 (23/113)	6.4 (9/157)
K14.K64-CRKP-pediatric (0% [0/18 isolates])							0 (0/1)	0 (0/3)	0 (0/12)	0 (0/2)
wzi209-CRKP-adult (0% [0/112 isolates])		0 (0/3)	0 (0/26)	0 (0/7)	0 (0/12)	0 (0/4)	0 (0/21)	0 (0/13)	0 (0/14)	0 (0/12)
wzi209-CRKP-pediatric (0% [0/314 isolates])				0 (0/1)	0 (0/7)	0 (0/2)	0 (0/16)	0 (0/76)	0 (0/77)	0 (0/135)
*rmpA2/terW*										
K14.K64-CRKP-adult (24.6% [129/533 isolates])					0 (0/14)	0 (0/50)	0 (0/63)	5.9 (8/136)	36.3 (41/113)	51.0 (80/157)
K14.K64-CRKP-pediatric (11.1% [2/18 isolates])							0 (0/1)	0 (0/3)	8.3 (1/12)	50.0 (1/2)
wzi209-CRKP-adult (2.7% [3/112 isolates])		0 (0/3)	0 (0/26)	0 (0/7)	0 (0/12)	0 (0/4)	0 (0/21)	15.4 (2/13)	0 (0/14)	8.3 (1/12)
wzi209-CRKP-pediatric (5.4% [17/314 isolates])				0 (0/1)	0 (0/7)	0 (0/2)	0 (0/16)	1.3 (1/76)	0 (0/77)	11.9 (16/135)

### Virulence plasmids Vir1 and Vir2 and Vir2-related fragment identified in *bla*_KPC_-carrying K14.K64-CRKP and wzi209-CRKP.

Three hv-CRKP strains, namely, XHKP502 (K14.K64-CRKP-adult-*rmpA*/*iroN*/*rmpA2*/*terW*), XHKPN083 (K14.K64-CRKP-adult-*rmpA2*/*terW*), and XHKPN391 (wzi209-CRKP-pediatric-*rmpA2*/*terW*), were randomly selected and sequenced with a PacBio Sequel system (Fig. 4B; also see Table S4). All three of these strains contained *bla*_KPC_-carrying IncFII-IncR. In addition, two virulence plasmids were identified only in XHKP502 and XHKPN083. The *bla*_KPC_-carrying IncFII-IncR plasmids within these three strains underwent distinct changes, compared to pXHKP6-1 (see Fig. S4A). Here, two different hybrid virulence plasmids, Vir1 and Vir2, were found. Vir1 was a nonconjugative IncFIB(k) (pCAV1099-114)-IncHI1B (pNDM-Mar) with *rmpA*/*iroN*/*rmpA2*/*terW*, as pXHKP502-1, and Vir2 was a conjugative antibiotic-resistant IncFIB (pNDM-Mar)-IncHI1B (pNDM-Mar) with *rmpA2*/*terW*, as pXHKPN083-1. In addition, a Vir2-related and chromosomally integrated *rmpA2*/*terW*-positive fragment was found in XHKPN391. Plasmid comparison showed that pXHKP502-1 was almost the same as pVir-CR-HvKP267, while pXHKPN083-1 showed high similarity to pHB25-1-vir (see Fig. S4B). The carriage of both antibiotic resistance genes, i.e., *aac(3)-IId*, *armA*, *bla*_DHA-1_, *bla*_SHV-12_, *bla*_TEM-B_, *mph*(A), *mph*(E), *msr*(E), *qnrB4*, and *sul1*, and two kinds of conjugation regions in pXHKPN083-1 endows isolates with antibiotic resistance, virulence, and transfer ability.

### Transmission of virulence plasmids and virulence-related elements in *bla*_KPC_-carrying K14.K64-CRKP and wzi209-CRKP.

**FIG 5:**
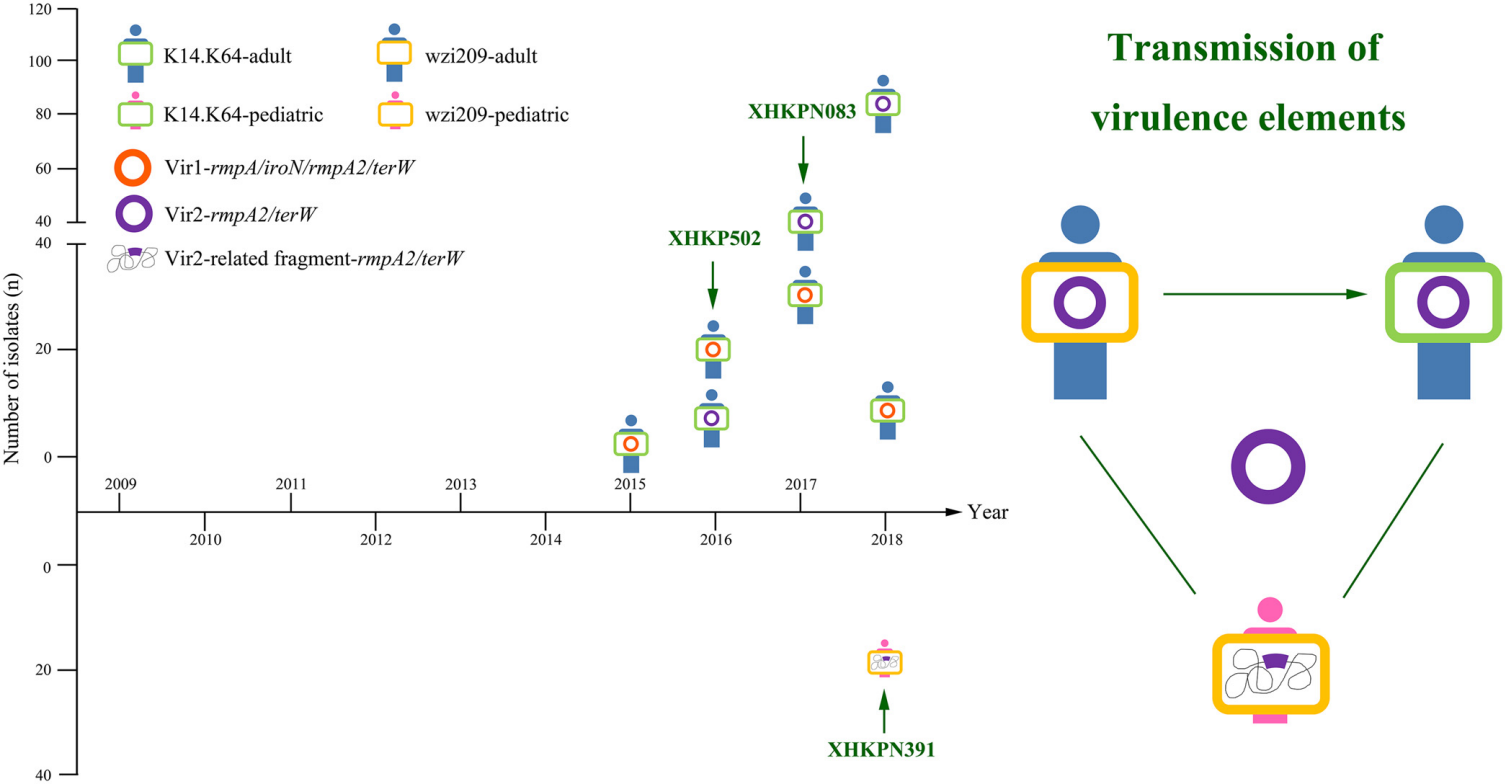
Transmission of virulence elements in *bla*_KPC_-carrying K14.K64-CRKP and wzi209-CRKP from 2009 to 2018. Vir1 with *rmpA/iroN/rmpA2/terW* is colored orange. Vir2 with *rmpA2/terW* is colored purple. Chromosomally integrated Vir2-related fragments are also shown. Strains carrying various virulence elements are shown. The isolation times of XHKP502, XHKPN083, and XHKPN391 are marked with green arrows.

As shown in Fig. 5, Vir1 emerged in 2014 and was disseminated and prevalent only among K14.K64-CRKP-adult strains (including XHKP502). Vir2 emerged in 2016 and was disseminated among K14.K64-CRKP-adult strains (including XHKPN083) with a higher prevalence rate. Notably, compared to Vir1, Vir2 showed a wider host range; it could be found in wzi209-CRKP-adult (XHKP585/XHKP588) and K14.K64-CRKP-pediatric (XHKPN097/XHKPN396) with low isolation rates and also in wzi209-CRKP-pediatric (XHKPN391) by chromosomal integration. To further confirm the chromosomally integrated condition found in wzi209-CRKP-pediatric, another three strains (XHKPN404, XHKPN469, and XHKPN494) were randomly selected and sequenced. Here, Vir2 with *rmpA2*/*terW* undoubtedly would be considered the most threatening plasmid.

### High stability and transmissibility of plasmids and hypervirulent phenotype for epidemic wzi209-CRKP-pediatric strains with Vir2-related fragment.

**FIG 6:**
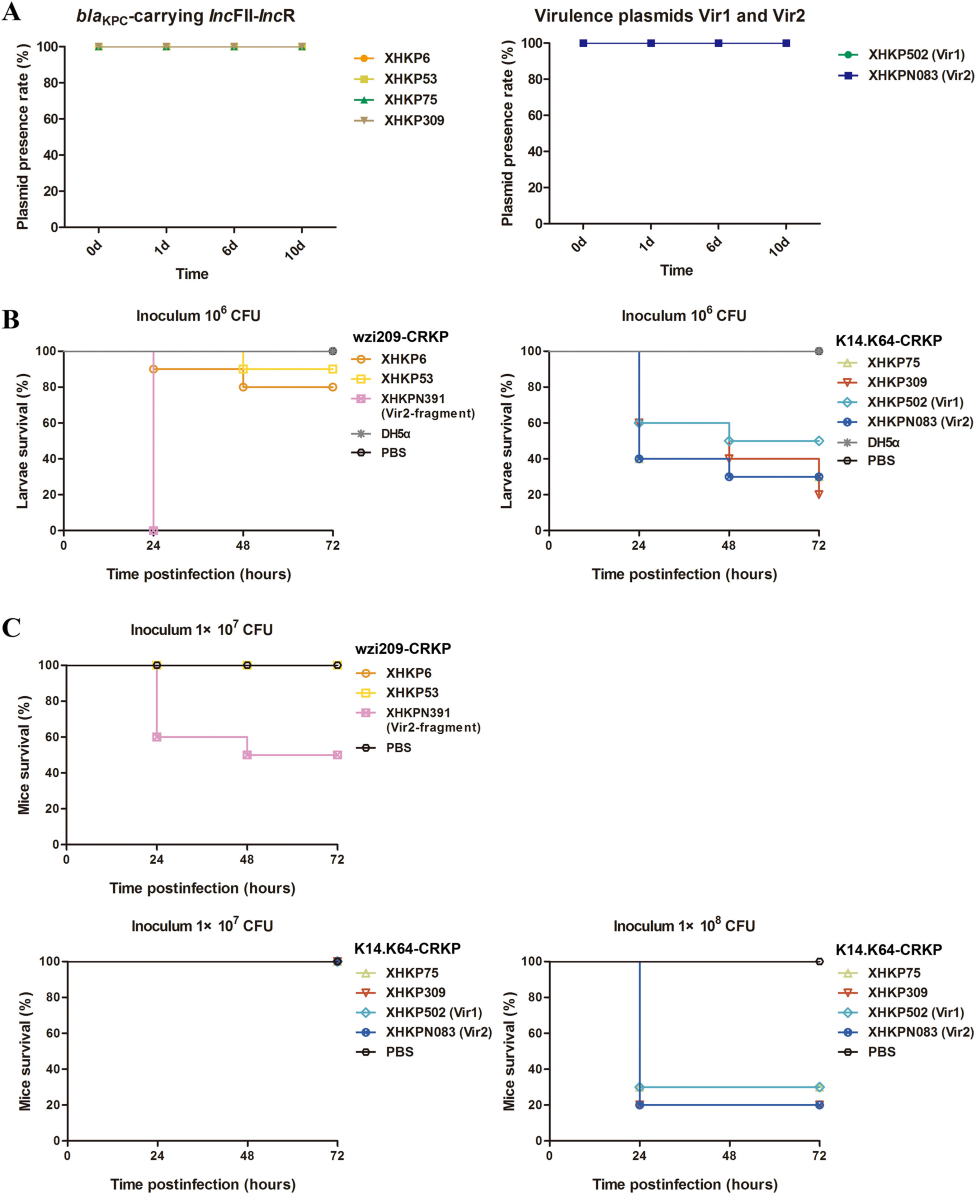
Plasmid stability and virulence characteristics of CRKP with distinct virulence elements. (A) Plasmids stability in CRKP and hv-CRKP. Plasmid stability of *bla*_KPC_-carrying IncFII-IncR was detected in XHKP6, XHKP53, XHKP75, and XHKP309 in LB medium. Plasmid stability of Vir1 and Vir2 was detected in XHKP502 and XHKPN083. (B) Virulence potential of wzi209-CRKP and K14.K64-CRKP strains in a Galleria mellonella larval infection model at 10^6^ CFU. As shown above, the survival of larvae (*n* = 10) infected by the indicated inoculum of each strain at 24 h, 48 h, and 72 h is shown. wzi209-CRKP group strains XHKP6, XHKP53, and XHKPN391 (Vir2 fragment) were included. K14.K64-CRKP group strains XHKP75, XHKP309, XHKP502 (Vir1), and XHKPN083 (Vir2) were also included. KPS53 (ST23/K1), which was isolated in our laboratory, was used as a positive control. DH5α cells and PBS were used as negative controls. Survival curves were plotted using the Kaplan-Meier method, and *P* values were calculated by the log rank (Mantel-Cox) test. A significant difference was observed with the 10^6^ CFU inoculum (*P < *0.0001 for XHKP6 versus XHKPN391 and *P < *0.0001 for XHKP53 versus XHKPN391), while no difference was observed within the K14.K64-CRKP group. (C) Virulence potential of strains belonging to the wzi209-CRKP and K14.K64-CRKP groups in a C57BL/6 mice infection model at 1 × 10^7^ and 1 × 10^8^ CFU. Strains were included as above. A significant difference was observed with the 10^7^ CFU inoculum (*P = *0.0167 for XHKP6 versus XHKPN391 and *P = *0.0167 for XHKP53 versus XHKPN391), while no difference was observed within K14.K64-CRKP groups for both the 10^7^ and 10^8^ CFU inocula. Each line represents a single isolate. Each experiment was repeated three times (*n* = 3). Representative experimental data are shown.

The plasmid stability of *bla*_KPC_-carrying IncFII-IncR and virulence plasmids Vir1 and Vir2 is shown in Fig. 6A. All of the plasmids exhibited high stability. As shown in Table 4, *bla*_KPC_-carrying IncFII-IncR in XHKP75 and XHKPN083 could conjugate to EC600 at rates of 1.38 × 10^−7^ and 3.33 × 10^−7^, respectively. The conjugation frequency of Vir2 in KPN083 was higher than that in IncFII-IncR, with a rate of 1.10 × 10^−5^. Coconjugation of both IncFII-IncR and Vir2 was also identified in XHKPN083, at a rate of 1.46 × 10^−7^. The *bla*_KPC_ and *rmpA2* genes were selected for detection of transconjugants.

**TABLE 4:** Phenotypic and genotypic characteristics of K. pneumoniae and transconjugants

Strain	Bacterial species	Capsular serotype	Plasmid	Conjugation ability^*a*^	Conjugation efficiency^*b*^	*bla*_KPC_	*rmpA2*
XHKP6	K. pneumoniae	wzi209	IncFII-IncR	−	ND	+	−
XHKP53	K. pneumoniae	wzi209	IncFII-IncR	−	ND	+	−
XHKP75	K. pneumoniae	K14.K64	IncFII-IncR	+	ND	+	−
XHKP309	K. pneumoniae	K14.K64	IncFII-IncR	−	ND	+	−
XHKP502	K. pneumoniae	K14.K64	IncFII-IncR/Vir1	−	ND	+	+
XHKPN083	K. pneumoniae	K14.K64	IncFII-IncR/Vir2	+	ND	+	+
EC600	E. coli	ND	ND	ND	ND	−	−
EC600-pXHKP75-2	E. coli	ND	IncFII-IncR	ND	1.38 × 10^−7^	+	−
EC600-pXHKPN083-2	E. coli	ND	IncFII-IncR	ND	3.33 × 10^−7^	+	ND
EC600-pXHKPN083-1	E. coli	ND	Vir2	ND	1.10 × 10^−5^	ND	+
EC600-pXHKPN083-1/2	E. coli	ND	IncFII-IncR/Vir2	ND	1.46 × 10^−7^	+	+

a −, negative; +, positive; ND, not done (the experiment or analysis was not performed with this strain).

b The ratio of transconjugants (EC600 with plasmids) to recipients (EC600).

Here, the hypervirulent phenotype was detected for strains with virulence elements in Galleria mellonella larval and murine infection models. K14.K64-CRKP (XHKP75, XHKP309, XHKP502, and XHKPN083) and wzi209-CRKP (XHKP6, XHKP53, and XHKPN391) were selected and used in this study. As shown in Fig. 6B and C, no significant difference was found for XHKP502 and XHKPN083, compared to XHKP75 and XHKP309, in K14.K64-CRKP by both Galleria mellonella larval and murine infection models, which suggested that the presence of virulence genes did not indicate the pathogenicity of strains. Surprisingly, the virulence of XHKPN391 was significantly higher than that of XHKP6 and XHKP53 in wzi209-CRKP, which may be related to the stable expression of virulence genes integrated into chromosomes. Further study is needed to determine the pathogenicity of XHKPN391-like isolates. Additionally, CRKP strains with and without virulence elements were not hypermucoviscous in this study (see Fig. S5).

## DISCUSSION

The global dissemination of CRKP has become a severe threat to public health, and the situation has worsened since the emergence of hv-CRKP. Despite the retrospective and single-center investigation, this study performed a 10-year systematic study to provide a uniquely clear and detailed picture of the whole process of antibiotic resistance and virulence, covering the early emergence of non-wzi209/K14.K64 CRKP to the epidemic CRKP and epidemic hv-CRKP. Two capsular types, K14.K64 and wzi209, were found. K14.K64-CRKP was found mainly in adult patients, especially patients >65 years of age, and wzi209-CRKP was found mainly in pediatric patients, especially infants, consistent with previous reports. The population bias for K14.K64 and wzi209 was unknown. K14.K64 is a more commonly observed capsule type than wzi209 and has been detected nationally (5, 23). In this study, both K14.K64-CRKP and wzi209-CRKP were widely disseminated.

The *bla*_KPC_-carrying hybrid plasmid IncFII-IncR was identified and contained two different parts. One part showed high similarity to pHN7A8, which was mainly prevalent in Klebsiella pneumoniae. Another part showed high similarity to IncR-related plasmids. Although the high stability of *bla*_KPC_-carrying IncFII-IncR was confirmed in pXHKP6-1, pXHKP53-1, pXHKP75-2, and pXHKP309-1, loss of genes or fragments in IncFII-IncR was observed later in pXHKP502-2 and pXHKPN083-2 during long-term activity. In this study, we found that conjugation regions on IncFII-IncR were variable over time, and they were successfully used as markers to track plasmid transmission. Therefore, we ascertained IncFII-IncR transmission among wzi209-CRKP-adult, wzi209-CRKP-pediatric, and K14.K64-CRKP-adult strains. However, the origin of IncFII-IncR in K14.K64-CRKP-pediatric strains was still unclear. The loss of conjugation ability increased genomic stability, which is reflected in the prevalence of *bla*_KPC_-carrying wzi209-CRKP over 10 years, but reduced the plasmid transmissibility to other strains. Moreover, we characterized the conjugation ability for *bla*_KPC_-carrying IncFII-IncR in XHKP75 but failed in XHKP6, XHKP53, and XHKP309, which implied that conjugation deficiency might have already occurred for pXHKP6-1 and pXHKP309-1. The virulence factors *iroN*, *terW*, *rmpA*, and *rmpA2* are well known in Klebsiella pneumoniae and were used to check for the presence of virulence elements. Two virulence forms, i.e., *rmpA*/*iroN*/*rmpA2*/*terW* and *rmpA2*/*terW*, were found in this study and were confirmed within all 977 *bla*_KPC_-carrying K14.K64-CRKP and wzi209-CRKP strains. The corresponding plasmids Vir1, i.e., nonconjugative IncFIB(k) (pCAV1099-114)-IncHI1B (pNDM-Mar), and Vir2, i.e., conjugative antibiotic-resistant IncFIB (pNDM-Mar)-IncHI1B (pNDM-Mar), were identified mainly in K14.K64-CRKP-adult strains. The coexistence of two virulence plasmids prevalent simultaneously within the same group of patients has rarely been reported previously. Vir1 had high similarity with the known virulence plasmid pLVPK, while Vir2 has rarely been reported thus far. As shown above, nonconjugative IncFII-IncR plasmids were widely disseminated in wzi209-CRKP-pediatric strains. Similarly, nonconjugative Vir1 plasmids also were prevalent in wzi209-CRKP-adult strains, which implied that there was no inevitable connection between the conjugation element and strain dissemination. However, a relatively higher prevalence of Vir2 than Vir1 was identified, which suggested the importance of the conjugation environment for transmission. Notably, Vir2 also contained drug resistance elements, which made this plasmid transferable, virulent, and antibiotic resistant among different *wzi*-type strains or strains of different species.

Here, we screened all 977 *bla*_KPC_-carrying IncFII-IncR strains and found that a large number of CRKP strains had virulence genes. Interestingly, neither Vir1 nor Vir2 enhanced the hypervirulent phenotype of CRKP, which was consistent with studies published recently. We discovered a group of CRKP strains, i.e., wzi209-CRKP-pediatric strains with a chromosomally integrated Vir2-related fragment, which exhibited extremely high virulence, compared to wzi209-CRKP strains. The hypervirulent phenotype was probably related to the high level of expression of virulence genes. Further study is needed in the future.

In conclusion, our findings described the antibiotic resistance and virulence of CRKP and hv-CRKP strains among adult and pediatric patients in a hospital setting. A large number of hv-CRKP strains was found. The dissemination of epidemic CRKP and hv-CRKP among patient groups has been clearly characterized to be mediated by plasmids and their derivatives. Hypervirulent wzi209-CRKP was generated with extremely enhanced virulence, compared to wzi209-CRKP with no virulence element, and can be considered a new threatening clone. Exploration among different hospitals and health care centers and worldwide surveillance and prevention are needed to monitor and control the spread of CRKP and hv-CRKP.

## MATERIALS AND METHODS

### Collection of strains and clinical information.

We performed a retrospective study in a 2,000-bed tertiary care hospital, with 1,400 beds for adult patients and 600 beds for pediatric patients, in China in 2009 to 2018. CRKP was defined as a clinical strain with nonsusceptibility to carbapenems (including imipenem, meropenem, and ertapenem), in accordance with the breakpoints in CLSI guidelines (26). A total of 1,181 nonduplicated CRKP strains were collected from inpatients in 2009 to 2018 during routine identification and antimicrobial susceptibility testing by the clinical laboratory of Xinhua Hospital, Shanghai Jiao Tong University School of Medicine (China). This retrospective study was approved by the research ethics board of Xinhua Hospital, Shanghai Jiao Tong University School of Medicine.

### Microbiological assessment.

Antimicrobial susceptibility testing of CRKP strains was conducted using the Vitek 2 Compact system combined with a disk diffusion method, and results were interpreted with CLSI criteria. The capsular polysaccharide serotypes of CRKP strains were determined by PCR and sequencing of the *wzi* allele (27). PCR products were sequenced and assigned using an online database (http://bigsdb.pasteur.fr/klebsiella). The *bla*_KPC_ gene was screened by PCR as described previously (28). Conjugation-related genes *traN* and *traC* were detected with primers designed in this study. Virulence-related genes *iroN*, *rmpA*, *terW*, and *rmpA2* were detected by PCR as an indication of virulence plasmids (29–31). All positive PCR products were sequenced and compared with sequences in GenBank (www.ncbi.nlm.nih.gov/blast). All primers are shown in Table S5 in the supplemental material. The hypermucoviscous phenotype was identified by string tests and sedimentation assays (32).

### Plasmid stability assay.

XHKP6, XHKP53, XHKP75, XHKP502, and XHKPN083 were recovered from −80°C storage. Three single colonies of each strain were picked and inoculated to fresh LB broth at 37°C with shaking (150 rpm) overnight. The cultures were diluted 1/1,000 into fresh LB broth every day. Aliquots were collected at 1, 6, and 10 days, serially diluted, and plated. Every 50 colonies were picked for three colonies of each strain and inoculated to clean water. Colony PCR was performed, and the presence of plasmids was detected by *bla*_KPC_, *rmpA2*, and *iroN* screening. PCR products were observed by electrophoresis and sequencing.

### Conjugation assay.

For both antibiotic-resistant and virulence plasmids, rifampin-resistant Escherichia coli strain EC600 was used as the recipient. Strains were cultured to logarithmic phase at 37°C in LB medium. Next, 1 mL of donor cells and 1 mL of recipient cells were mixed and inoculated. After incubation overnight, bacteria were collected and serially diluted. The diluted culture was spread on plates containing 4 μg/mL meropenem and 600 μg/mL rifampin to detect *bla*_KPC_-carrying IncFII-IncR. Plates containing 8 μg/mL sodium tellurite (Na_2_TeO_3_) and 600 μg/mL rifampin were used for virulence plasmids. The presence of *bla*_KPC_ and *rmpA2* as marker genes of plasmids in transconjugants was determined by PCR. To calculate the conjugation efficiency, the diluted culture was spread on plates containing only 600 μg/mL rifampin to determine the total number of recipient cells. The conjugation efficiency was calculated by dividing the number of transconjugants by the number of recipient cells.

### Infection models.

Pathogenicity was assessed in Galleria mellonella larvae and C57BL/6 mice. Larvae weighing approximately 300 mg (purchased from Tianjin Huiyude Biotech Co., Tianjin, China) were used as described previously (33, 34). Overnight cultures were washed with phosphate-buffered saline (PBS) and further adjusted with PBS to concentrations of 1 × 10^6^ CFU/mL. Ten G. mellonella larvae in each group were infected with bacteria in a 10-μL inoculum and incubated at 37°C. The survival rate of the G. mellonella larvae was recorded for 72 h. Three experiments were repeated. Female C57BL/6 mice (average weight of ~20 g, 5 weeks of age) were purchased from the Jihui Laboratory Animal Breeding Ltd. Co. (Shanghai, China). Mice were randomly allocated into different groups, with 10 mice per group. Mice in each group were infected intraperitoneally with 1.0 × 10^7^ CFU or 1.0 × 10^8^ CFU of each K. pneumoniae strain tested. The mortality rate of the test mice was observed and recorded for 72 h postinfection. Survival curves were generated using Prism. Statistical analysis was performed using the log rank (Mantel-Cox) test. All animal experiments were carried out in accordance with the recommendations in the guidelines of the institutional animal care and use committee of Xin Hua Hospital.

### Whole-genome sequencing and analysis.

Four CRKP isolates (XHKP6, XHKP53, XHKP75, and XHKP309) that first emerged in each group were subjected to long-read genome sequencing using the PacBio RS II system. *De novo* assembly was performed using HGAP3 of SMRT Link v2.3. Three hv-CRKP strains of the epidemic group (XHKP502, XHKPN083, and XHKPN391) were sequenced using the PacBio Sequel system. *De novo* assembly was performed using HGAP4 of SMRT Link v6. Gene prediction was performed using GLIMMER v3, and functional annotation was performed with BLASTp against the NCBI nonredundant peptide database, with parameters set at an E value of 1e−5. Clusters of Orthologous Groups (COG) assignment was predicted through RPS-BLAST with the Conserved Domain Database (CDD) at an E value of 1e−5. In addition, strains that emerged in K14.K64-CRKP-pediatric (XHKPN097 and XHKPN396) and wzi209-CRKP-adult (XHKP585 and XHKP588) samples were also sequenced using an Illumina X Ten instrument with 2 × 150-bp paired-end read libraries. In addition, to confirm the condition of virulence-related fragments integrated into chromosomes in wzi209-CRKP-pediatric strains, another three isolates (XHKPN404, XHKPN469, and XHKPN494) were sequenced with the Illumina X Ten system. *De novo* assembly of the short-read data was performed using Velvet v1.2.06 after quality trimming (Phred quality scores of >20) and adapter trimming by Trimmomatic v0.32. The ortholog clusters were identified by PGAP; the parameters are coverage of 0.5 and identity of 0.5. Plasmid replication type was confirmed by PlasmidFinder (https://cge.cbs.dtu.dk/services/PlasmidFinder). Antibiotic resistance genes within assembled genome sequences were identified using ResFinder 4.0 (https://cge.cbs.dtu.dk/services/ResFinder) (35). The insertion sequences were obtained by ISfinder (https://www-is.biotoul.fr). Single-nucleotide polymorphisms (SNPs) were identified by MUMmer.

### Data availability.

We deposited the whole-genome sequences of seven CRKP isolates in GenBank under the following accession numbers: XHKP6, CP066887, CP066888, CP066889, and CP066890; XHKP53, CP066891, CP066892, CP066893, and CP066894; XHKP75, CP066895, CP066896, CP066897, CP066898, and CP066899; XHKP309, CP066900, CP066901, CP066902, and CP066903; XHKP502, CP066904, CP066905, CP066906, CP066907, CP066908, and CP066909; XHKPN083, CP066910, CP066911, CP066912, CP066913, and CP066914; XHKPN391, CP066915, CP066916, CP066917, and CP066918. The draft genome sequences of the other seven CRKP isolates are also available, under the following accession numbers: XHKP585, JAEOAJ000000000; XHKP588, JAEOAK000000000; XHKPN097, JAEOAL000000000; XHKPN396, JAEOAM000000000; XHKPN404, JAEOAN000000000; XHKPN469, JAEOAO000000000; XHKPN494, JAEOAP000000000.
