# Supplementary material for: Cooccurrence of Antibiotic Resistance and Hypervirulence in High-Risk Carbapenem-Resistant K14.K64 and Wzi209 Klebsiella pneumoniae Strains Driven by Plasmids and Their Derivatives
Source: Microbiol Spectr. 2022 Aug 22;10(5):e02541-21. doi: 10.1128/spectrum.02541-21 (PMC9603693; doi:10.1128/spectrum.02541-21)
Supplement: Supplemental file 1 — Supplemental material. Download spectrum.02541-21-s0001.pdf, PDF file, 1.9 MB [file spectrum.02541-21-s0001.pdf]

Co-occurrence of antibiotic resistance and hypervirulence in high-risk carbapenem-resistant K14.K64 and Wzi209 *Klebsiella pneumoniae* driven by plasmids and their derivatives

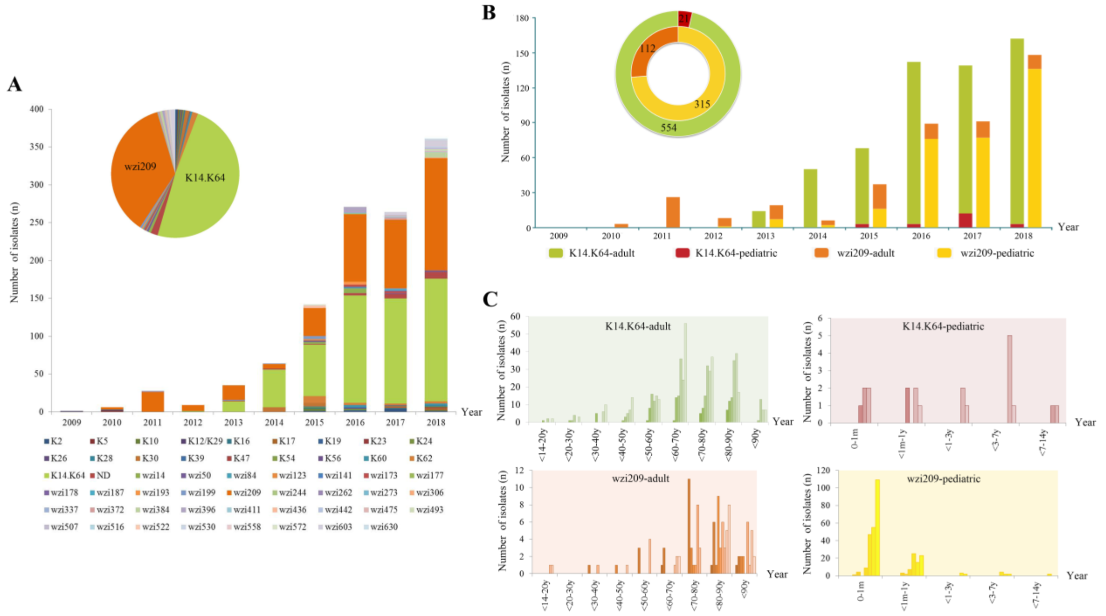

Fig. S1. The distribution of CRKP by serotype (*wzi*) sequencing from 2009 to 2018, including 1181 CRKP isolates collected. (A) Annual distribution of the overall capsule types of all CRKP isolates. Wzi209 was colored orange and K14.K64 was colored green. The x-coordinate was the year and the y-coordinate was the number of isolates in each year. (B) Age distribution of patients with both wzi209-CRKP and K14.K64-CRKP isolation. Wzi209-CRKP isolated from adult (wzi209-adult) was colored orange; wzi209-CRKP isolated from pediatric (wzi209-pediatric) was colored yellow; K14.K64-CRKP isolated from adult (K14.K64-adult) was colored red; K14.K64-CRKP isolated from pediatric (K14.K64-pediatric) was colored green. The x-coordinate was different age groups, and the y-coordinate was the number of isolated strains. (C) Annual distribution of

wzi209-adult, wzi209-pediatric, K14.K64-adult, and K14.K64-pediatric. Wzi209-adult was colored orange, wzi209-pediatric was colored yellow, K14.K64-adult was colored green and K14.K64-pediatric was colored red.

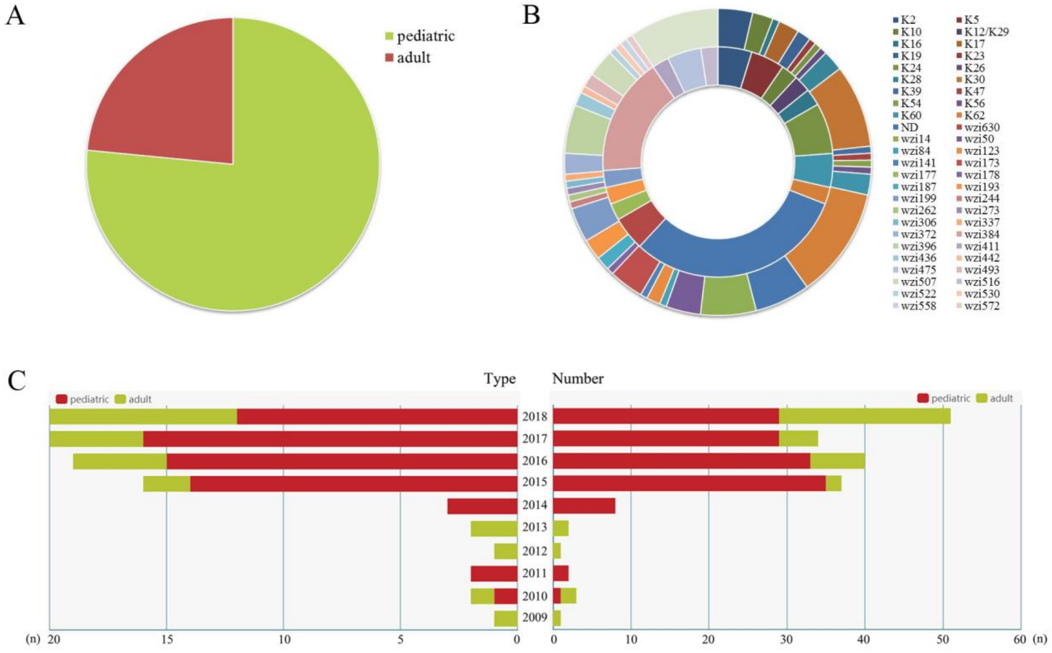

Fig. S2. Characteristics of 179 non-K14.K64/wzi209 CRKP strains isolated from 2009 to 2018. (A) The overall proportion of CRKP isolated from both pediatric and adult. (B) The capsular type distribution of non-K14.K64/wzi209 CRKP strains by *wzi* typing. (C) The accumulated capsule types and amount in adult and pediatric. CRKP isolated from adult patients were colored red and CRKP isolated from pediatric patients were colored green.

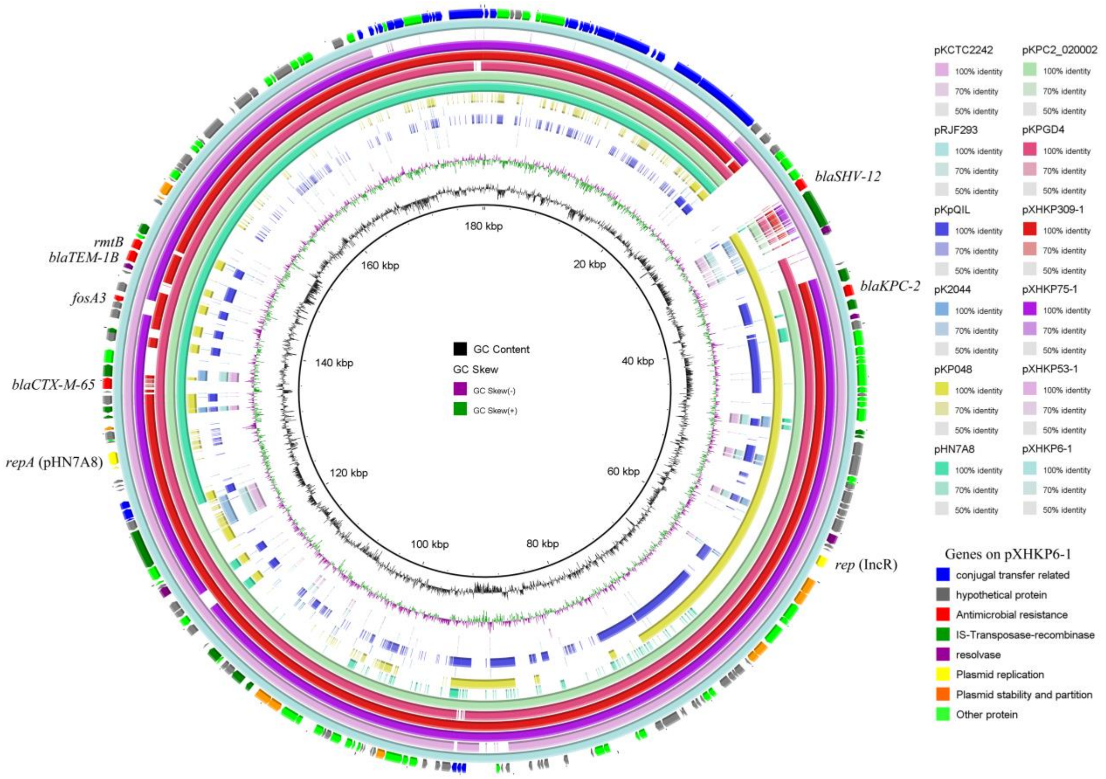

Fig. S3. Sequences alignment analysis of plasmids. pXHKP6-1, pXHKP53-1, pXHKP75-2, pXHKP309-1 and previously reported *bla*$_{\text{KPC-2}}$ carrying plasmids pKPGD-4 (NZ_CP025952.1), pKPC2_020002 (CP028541.2), pHN7A8 (JN232517.1), pKP048 (FJ628167.2), pK2044 (NC_006625.1), pKPQIL (GU595196.1), pRJF293 (CP014009.1), and pKCTC2242 (NC_017541.1) were shown here. Similarity was shown in depth of colors. *bla*$_{\text{KPC}}$-surrounded antibiotic resistance region and conjugation region are marked in red and blue, respectively. The outermost circle was the *orf*s display of pXHKP6-1. Resistance genes on the outermost circle are in red, and *rep* genes are in yellow.

A

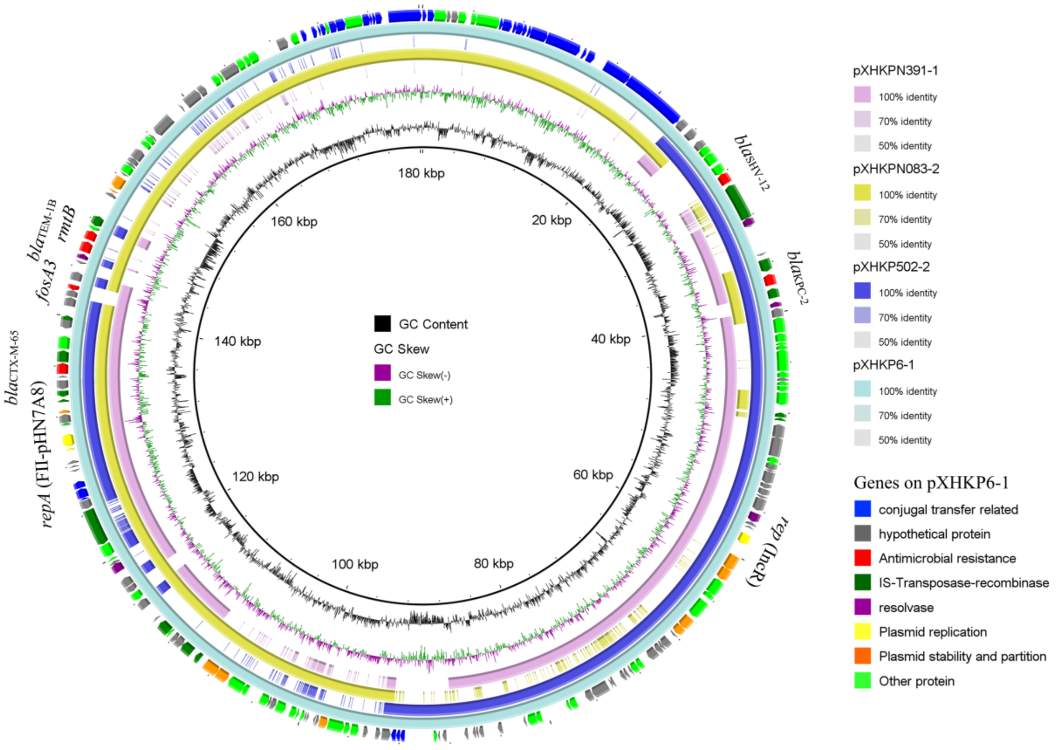
180 kbp
20 kbp
40 kbp
60 kbp
80 kbp
100 kbp
120 kbp
140 kbp
160 kbp
GC Content
GC Skew
GC Skew(-)
GC Skew(+)
pXHKPN391-1
100% identity
70% identity
50% identity
pXHKPN083-2
100% identity
70% identity
50% identity
pXHKP502-2
100% identity
70% identity
50% identity
pXHKP6-1
100% identity
70% identity
50% identity
Genes on pXHKP6-1
conjugal transfer related
hypothetical protein
Antimicrobial resistance
IS-Transposase-recombinase
resolvase
Plasmid replication
Plasmid stability and partition
Other protein
blaSHV-12
blaKPC-2
rep (IncR)
blaTEM-1B
rmtB
fosA3
blaCTX-M-65
repA (FII-pHN7A8)

B

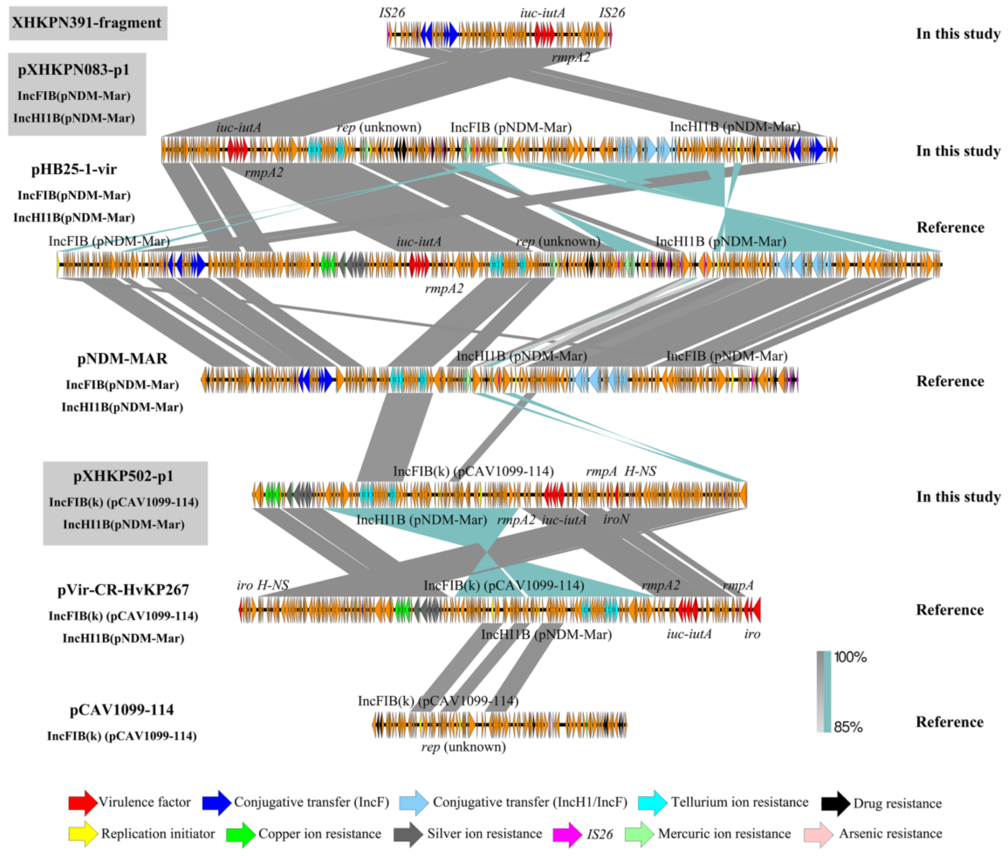
XHKPN391-fragment
IS26
iuc-iutA
IS26
rmpA2
In this study
pXHKPN083-p1
IncFIB(pNDM-Mar)
IncHI1B(pNDM-Mar)
iuc-iutA
rep (unknown)
IncFIB (pNDM-Mar)
IncHI1B (pNDM-Mar)
rmpA2
In this study
pHB25-1-vir
IncFIB(pNDM-Mar)
IncHI1B(pNDM-Mar)
IncFIB (pNDM-Mar)
iuc-iutA
rep (unknown)
IncHI1B (pNDM-Mar)
rmpA2
Reference
pNDM-MAR
IncFIB(pNDM-Mar)
IncHI1B(pNDM-Mar)
IncHI1B (pNDM-Mar)
IncFIB (pNDM-Mar)
Reference
pXHKP502-p1
IncFIB(k) (pCAV1099-114)
IncHI1B(pNDM-Mar)
IncFIB(k) (pCAV1099-114)
rmpA H-NS
IncHI1B (pNDM-Mar)
rmpA2 iuc-iutA
iroN
In this study
pVir-CR-HvKP267
IncFIB(k) (pCAV1099-114)
IncHI1B(pNDM-Mar)
iro H-NS
IncFIB(k) (pCAV1099-114)
rmpA2
rmpA
IncHI1B (pNDM-Mar)
iuc-iutA
iro
Reference
100%
85%
pCAV1099-114
IncFIB(k) (pCAV1099-114)
IncFIB(k) (pCAV1099-114)
rep (unknown)
Reference
Virulence factor
Conjugative transfer (IncF)
Conjugative transfer (IncH1/IncF)
Tellurium ion resistance
Drug resistance
Replication initiator
Copper ion resistance
Silver ion resistance
IS26
Mercuric ion resistance
Arsenic resistance

Fig. S4. Plasmid comparison of *bla*$_{KPC-2}$ carrying *Inc*FII-*Inc*R and virulence elements. (A) Sequence alignment analysis of *bla*$_{KPC-2}$ carrying *Inc*FII-*Inc*R (pXHKP6-1, pXHKP502-2, pXHKP083-2, and pXHKP391-1). The outermost circle was the *orf*s display of pXHKP6-1. Resistance genes on the outermost circle, including *bla*$_{KPC-2}$, *bla*$_{SHV-12}$, *bla*$_{CTX-M-65}$, *fosA3, bla*$_{TEM-1B}$, and *rmtB*, were colored red. *rep* genes were colored yellow. (B) Sequence alignment analysis of pXHKP502-1, pXHKP083-1, virulence fragment on pXHKP391, virulence plasmids pHB25-1-vir (CP039526.1) and pVir-CR-HvKP267 (MG053312.1), antibiotic resistance plasmids pNDM-MAR (JN420336.1) and pCAV1099-114 (CP011596.1). Plasmid genes were marked and colored.

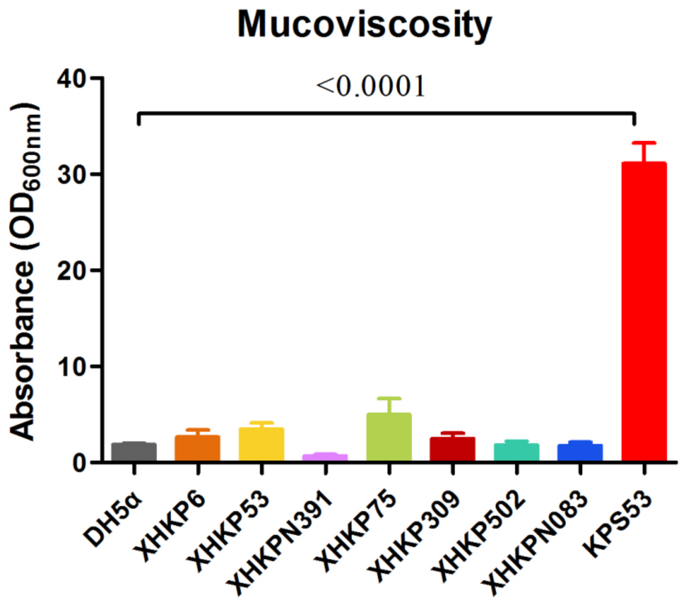

Fig. S5. Mucoviscosity of *bla*KPC carrying CRKP with and without virulence elements. XHKP6, XHKP53, XHKP75, XHKP309, XHKP502, XHKPN083 and XHKPN391 were included. KPS53 (ST23/K1) isolated in our lab was used as a positive control. DH5α was used as negative control. The mucoviscosity determined by centrifugation was represented by the OD600 of three independent experiments. The absorbance percentage was shown. Each experiment was repeated three times ($n = 3$). One-way ANOVA was performed for statistical analyses. Data are presented as the mean ± s.e.m.

Table S1. Age distribution of inpatients with CRKP isolation from 2009-2018.

	K14.K64 (n=575)	wzi209 (n=427)	non-K14.K64/wzi209 (n=179)
**Adult >14y (n=708, 60.0%), n**	554	112	42
>14, ⩽20y (n=7, 1.0%), n (%, %)	5 (0.9, 71.4)	2 (1.8, 28.6)	0
>20, ⩽30y (n=10, 1.4%), n (%, %)	9 (1.6, 90.0)	0	1 (2.4, 10.0)
>30, ⩽40y (n=23, 3.3%), n (%, %)	21 (3.8, 91.3)	2 (1.8, 8.7)	0
>40, ⩽50y (n=42, 5.9%), n (%, %)	30 (5.4, 71.4)	2 (1.8, 4.8)	10 (23.8, 23.8)
>50, ⩽60y (n=76, 10.7%), n (%, %)	65 (11.7, 85.5)	7 (6.3, 9.2)	4 (9.5, 5.3)
>60, ⩽70y (n=164, 23.2%), n (%, %)	146 (26.4, 89.0)	9 (8.0, 5.5)	9 (21.4, 5.5)
>70, ⩽80y (n=157, 22.2%), n (%, %)	126 (22.7, 80.3)	27 (24.1, 17.2)	4 (9.5, 2.6)
>80, ⩽90y (n=178, 25.1%), n (%, %)	124 (22.4, 69.7)	42 (37.5, 23.6)	12 (28.6, 6.7)
>90y (n=51, 7.2%), n (%, %)	28 (5.1, 54.9)	21 (18.8, 41.2)	2 (4.8, 3.9)
**Pediatric ⩾0, ⩽14 (n=473, 40.05%), n**	21	315	137
>0, ⩽1m (302, 63.9%), n (%, %)	5 (23.8, 1.7)	225 (71.4, 74.5)	72 (52.6, 23.8)
>1m, ⩽1y (135, 28.5%), n (%, %)	5 (23.8, 3.7)	75 (23.8, 55.6)	55 (40.2, 40.7)
>1, ⩽3y (15, 3.2%), n (%, %)	3 (14.3, 20.0)	5 (1.6, 33.3)	7 (5.1, 46.7)
>3, ⩽7y (16, 3.4%), n (%, %)	6 (28.6, 37.5)	8 (2.5, 50.0)	2 (1.5, 12.5)
>7, ⩽14y (5, 1.1%), n (%, %)	2 (9.5, 40.0)	2 (0.6, 40.0)	1 (0.7, 20.0)

Table S2. General genomic features of XHKP6, XHKP53, XHKP75, and XHKP309.

	XHKP6	XHKP53	XHKP75	XHKP309
**Size, bp**	5,645,164	5,572,604	5,868,873	5,633,251
**G+C content**	57.3	57.3	57.1	57.3
**No. of CDS**	5,493	5,516	5,690	5,465
**rRNA,n**	25	25	25	25
**tRNA, n**	85	85	85	85
**Plasmids, n**	3	3	4	3
**Prophages, n**	14	15	13	12
**IS elements, n**	52	57	72	64
TnAs3	2	2	3	2
ISEcp1	2	2	2	2
ISCfr1	1	1	1	1
ISKpn1	5	5	7	7
ISKpn18	1	2	1	1
ISKpn26	13	12	16	20
ISKpn28	1	1	1	1
IS26	14	15	16	17
ISKpn14	4	6	7	4
TnAs1	1	1	0	0
Tn2	1	1	1	1
IS5075	2	3	5	3
ISCfr3	1	1	1	1
ISKpn27	1	1	1	2
ISKpn6	1	1	1	2
IS1294	1	1	1	0
IS903	1	2	3	0
Tn5403	0	0	1	0
ISKpn33	0	0	1	0
ISEcl1	0	0	1	0
ISKpn38	0	0	1	0
IS6100	0	0	1	0

Table S3. Prevalence of virulence genes in *bla*$_{KPC-2}$ carrying K14.K64-CRKP and wzi209-CRKP within adult and pediatric patients isolated from 2009-2018.

Virulence genes	2009	2010	2011	2012	2013	2014	2015	2016	2017	2018
rmpA										
K14.K64-adult (9.4%, 50/533)					0%(0/14)	0%(0/50)	**3.2%(2/63)**	**11.0%(15/136)**	**20.4%(23/113)**	**6.4%(10/157)**
K14.K64-pediatric (0%, 0/18)							0%(0/1)	0%(0/3)	0%(0/12)	0%(0/2)
Wzi209-adult (0%, 0/112)		0%(0/3)	0%(0/26)	0%(0/7)	0%(0/12)	0%(0/4)	0%(0/21)	0%(0/13)	0%(0/14)	0%(0/12)
Wzi209-pediatric (0%, 0/314)				0%(0/1)	0%(0/7)	0%(0/2)	0%(0/16)	0%(0/76)	0%(0/77)	0%(0/135)
iroN										
K14.K64-adult (9.6%, 51/533)					0%(0/14)	0%(0/50)	**3.2%(2/63)**	**11.0%(15/136)**	**20.4%(23/113)**	**7.0%(11/157)**
K14.K64-pediatric (0%, 0/18)							0%(0/1)	0%(0/3)	0%(0/12)	0%(0/2)
Wzi209-adult (0%, 0/112)		0%(0/3)	0%(0/26)	0%(0/7)	0%(0/12)	0%(0/4)	0%(0/21)	0%(0/13)	0%(0/14)	0%(0/12)
Wzi209-pediatric (0%, 0/314)				0%(0/1)	0%(0/7)	0%(0/2)	0%(0/16)	0%(0/76)	0%(0/77)	0%(0/135)
rmpA2										
K14.K64-adult (33.8%, 180/533)					0%(0/14)	0%(0/50)	**3.2%(2/63)**	**16.9(23/136)**	**56.6%(64/113)**	**58.0%(91/157)**
K14.K64-pediatric (11.1%, 2/18)							0%(0/1)	0%(0/3)	**8.3%(1/12)**	**50.0%(1/2)**
Wzi209-adult (2.7%, 3/112)		0%(0/3)	0%(0/26)	0%(0/7)	0%(0/12)	0%(0/4)	0%(0/21)	**15.4%(2/13)**	0%(0/14)	**8.3%(1/12)**
Wzi209-pediatric (5.7%, 18/314)				0%(0/1)	0%(0/7)	0%(0/2)	0%(0/16)	**2.6%(2/76)**	0%(0/77)	**11.9%(16/135)**
terW										
K14.K64-adult (33.6%, 179/533)					0%(0/14)	0%(0/50)	**3.2%(2/63)**	**16.9(23/136)**	**56.6%(64/113)**	**57.3%(90/157)**
K14.K64-pediatric (11.1%, 2/18)							0%(0/1)	0%(0/3)	**8.3%(1/12)**	**50.0%(1/2)**
Wzi209-adult (3.6%, 4/112)		0%(0/3)	0%(0/26)	0%(0/7)	0%(0/12)	0%(0/4)	0%(0/21)	**23.1%(3/13)**	0%(0/14)	**8.3%(1/12)**
Wzi209-pediatric (5.7%, 18/314)				0%(0/1)	0%(0/7)	0%(0/2)	0%(0/16)	**2.6%(2/76)**	0%(0/77)	**11.9%(16/135)**

Table S4. General genomic features of XHKP502, XHKPN083, and XHKPN391.

	XHKP502	XHKPN083	XHKPN391
**Size, bp**	5,887,904	5,804,025	5,763,410
**G+C content,**	57.0	56.8	57.2
**No. of CDS**	5,715	5,540	5,614
**rRNA,n**	25	25	25
**tRNA, n**	86	85	86
**Plasmids, n**	5	4	3
**Prophages, n**	13	13	11
**IS elements, n**	74	82	79
TnAs3	3	1	1
ISEcp1	2	4	2
ISCfr1	1	2	1
ISKpn1	7	7	4
ISKpn18	2	1	1
ISKpn26	21	13	22
ISKpn28	1	5	1
IS26	11	16	22
ISKpn14	3	3	5
TnAs1	2	0	2
Tn2	1	2	2
IS5075	6	5	2
ISCfr3	0	1	1
ISKpn27	1	1	3
ISKpn6	1	1	3
IS1294	1	2	2
IS903	2	7	1
IS6100	0	1	0
ISShes11	1	0	0
ISKox1	1	0	0
ISKpn42	1	0	0
ISKpn54	1	0	0
IS1A	3	1	1
ISKpn19	1	0	0
ISVsa3	1	0	0
ISKpn21	0	2	1
ISEc29	0	1	0
ISKpn41	0	2	0
ISEc52	0	2	2
ISEc33	0	1	0
ISEc28	0	1	0

Table S5. Primers used in this study.

	Sequence (5'-3')
wzi	F: GTGCCGCGAGCGCTTTCTATCTTGGTATTCC
	R: GAGAGCCACTGGTTCCAGAAYTTSACCGC
***bla*KPC**	F：GCTACACCTAGCTCCACCTTC
	R：ACAGTGGTTGGTAATCCATGC
traN	F: GC TAACGGTAAC TGTCTTTCT
	R: CTGCCAAACCCGATACGCAACT
traC	F: GATTACAAC CAGGAATCCG GTCT
	R: GCTGGTTACGGTAGGTGGGT
rmpA	F: ACTGGGCTACCTCTGCTTCA
	R: CTTGCATGAGCCATCTTTCA
rmpA2	F: TGTGCAATAAGGATGTTACATTAGT
	R: TTTGATGTGCACCATTTTTCA
iroN	F: AAGTCAAAGCAGGGGTTGCCCG
	R:GACGCCGACATTAAGACGCAG
terW	F: ATGCAATTAAACACCAGACAG
	R:CTCATTCTCTTGAGTGTTTTC
